# Lipoblastoma-Like Tumor: A Case Report From Costa Rica

**DOI:** 10.7759/cureus.94569

**Published:** 2025-10-14

**Authors:** Adrian Castro Madrigal, Pedro Madriz de Haan, Sofía Suárez Sánchez

**Affiliations:** 1 Gynecologic Oncology, Hospital Clínica Bíblica, San Jose, CRI; 2 Pathology, Hospital Rafael Ángel Calderón Guardia, San Jose, CRI; 3 Life Science Center for Innovation, Hospital Clínica Bíblica, San Jose, CRI

**Keywords:** adipocytic neoplasm, lipoblastoma, myxoid stroma, spindle cell, vulva

## Abstract

Lipoblastoma-like tumors (LLTs) are rare adipocytic neoplasms, most commonly found in the vulvar region. Their histopathological and molecular features often overlap with other adipocytic tumors, making diagnosis challenging. We report the case of a 41-year-old woman who presented with a painless mass in the right labia majora. An initial excisional biopsy confirmed a lipoblastoma-like tumor, characterized by spindle cells in a myxoid stroma, focal positivity for CD34 and S100, and absence of DDIT3 rearrangement. Imaging revealed residual nodular lesions, prompting a wide local excision, which achieved negative margins. No recurrence was observed during a 24-month follow-up. LLTs pose diagnostic challenges due to their morphological similarities with lipoblastoma, myxoid liposarcoma, spindle cell lipoma, and atypical spindle cell/pleomorphic lipomatous tumor (ASCPLT). Immunohistochemical and molecular analyses, especially the absence of DDIT3 and PLAG1 rearrangements, are essential for accurate diagnosis. Surgical excision with clear margins remains the treatment of choice, and long-term monitoring is advised due to the potential, though rare, risk of recurrence or metastasis. LLTs should be considered in the differential diagnosis of vulvar soft tissue masses. Accurate histopathological and molecular evaluation, combined with appropriate surgical management, ensures favorable outcomes and helps avoid unnecessary interventions.

## Introduction

Lipoblastoma-like tumors (LLTs) were first described by Lae et al. [1] as uncommon adipocytic neoplasms of the vulva. Less than 30 cases have been reported in the literature [2]. These neoplasms display morphological characteristics distinct from both lipoblastomas and liposarcomas. Histologically, they consist of spindle cells in a myxoid stroma with prominent vasculature and variable adipocytic differentiation. At the molecular level, they are characterized by the absence of PLAG1 and HMGA2 expression and by the lack of DDIT3 rearrangements, as detected by fluorescence in situ hybridization (FISH), which distinguishes them from lipoblastomas and myxoid liposarcomas [3-5]. The clinical relevance of recognizing this entity lies in its distinction from myxoid liposarcoma, a malignant tumor with very different management and prognosis. Accurate diagnosis of LLT avoids overtreatment, unnecessary radical surgery, or adjuvant therapy, ensuring that patients receive conservative but adequate surgical management with long-term follow-up [5].

However, there is no consensus regarding their molecular profile, as discrepancies have been reported in the expression of various markers such as RB1, CD34, and S100. Cases with CD34+ and S100+ expression, as well as S100- and occasionally CD34-, have been described. This variability can complicate the differential diagnosis with other lipomatous tumors, particularly spindle cell lipoma, atypical spindle cell/pleomorphic lipomatous tumor (ASCPLT), and lipoblastoma, which share overlapping immunohistochemical profiles [3].

LLTs are generally negative for MDM2 and CDK4, though focal positivity has been observed in rare cases [2]. The 2020 WHO diagnostic criteria for LLTs include both essential and desirable features. Essential criteria consist of a lobulated growth pattern demarcated by fibrous septa of variable thickness, the presence of a mixture of mature adipocytes, univacuolated and bivacuolated lipoblasts, and bland spindle cells, along with abundant myxoid matrix and a thin-walled branching vasculature. Desirable criteria include the absence of DDIT3 or PLAG1 rearrangements, as well as RB1 gain or loss detected by FISH, and absence of PLAG1 and HMGA2 expression in immunohistochemical studies. These criteria help distinguish LLT from other adipocytic neoplasms and highlight the importance of histopathological, immunohistochemical, and molecular evaluation in their diagnosis [6].

## Case presentation

**Figure 1:**
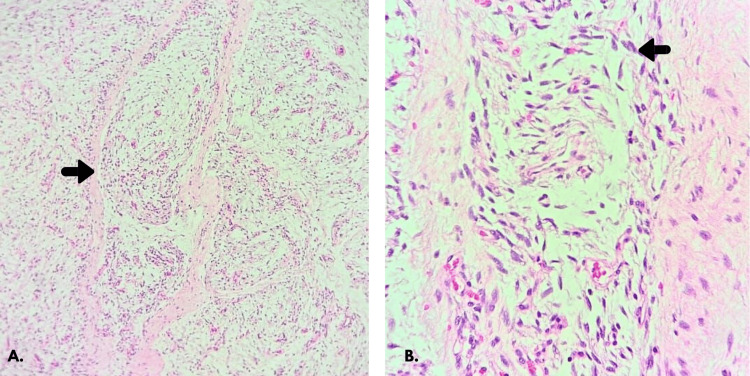
Histopathological features of the patient’s vulvar LLT biopsy. (A) Lobulated adipocytic tissue with fibrous septa (arrow). (B) Spindle cell within a myxoid stroma (arrow). LLT: lipoblastoma-like tumor.

A 41-year-old female patient, nulliparous, with no relevant medical or surgical history, and a user of combined oral contraceptives, presented with a sensation of a mass arising from the upper third of the right labia majora, initially detected on clinical palpation and subsequently confirmed by ultrasound. She reported no additional symptoms. An excisional biopsy was performed in an outpatient setting, yielding an elongated translucent cystic structure partially covered by fibroadipose tissue, measuring 3.2 × 2 × 1.4 cm. On sectioning, it contained myxoid-like fluid. Histopathology revealed a LLT with involved margins. Immunohistochemistry showed focal positivity for CD34, S100, and vimentin, weak focal positivity for ER, and negativity for SOX-10, PanCK, EMA, GFAP, PR, smooth muscle actin, and PanTRK. Histological examination described a lobulated lesion composed of spindle cells in a myxoid stroma with prominent vasculature, without atypia, mitoses, or necrosis (Figure 1A, B). Cytogenetic analysis revealed no DDIT3 rearrangement and no RB1 loss.

**Figure 2:**
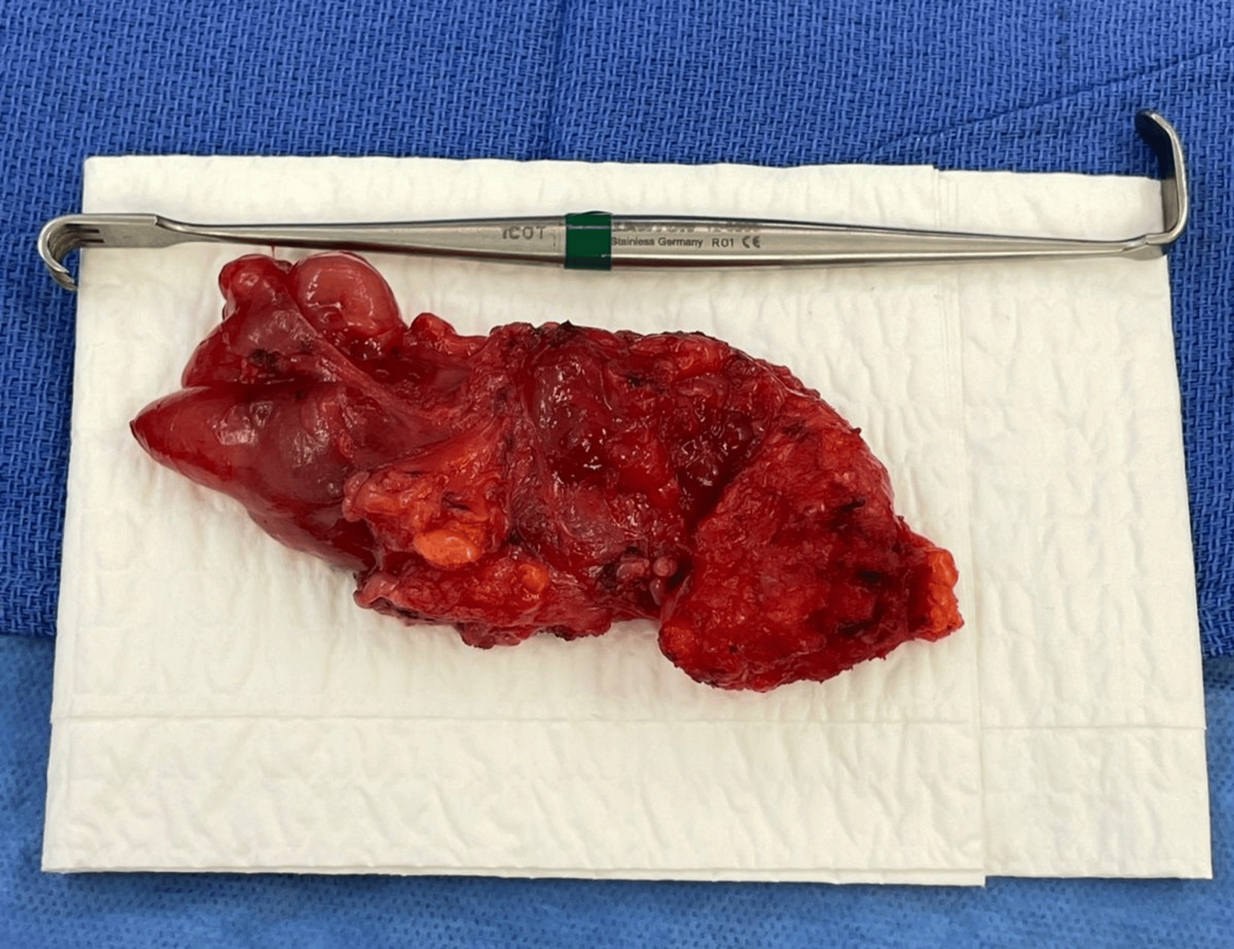
LLT extracted during right inguinal-femoral local surgical resection. LLT: lipoblastoma-Like tumor.

A follow-up ultrasound performed three months later identified solid, oval, heterogeneous nodules in the right inguinofemoral region, measuring up to 57 × 15 mm, with Doppler showing vascularization. A subsequent CT scan revealed two homogeneous hypodense nodules in the right inguinal canal, measuring 31 mm and 23 mm, without evidence of disease elsewhere. A wide local inguinovulvar resection was performed (Figure 2), extending to the deep fascia, and the specimen was submitted with orientation for pathological evaluation to better assess surgical margins. Histopathology confirmed the same neoplasm previously diagnosed, described as a proliferation of spindle cells in a myxoid stroma without atypia, mitoses, or necrosis. The resection specimen measured up to 10.5 × 4.5 × 2 cm, with the largest nodule measuring 3 × 4.5 cm. Margins were initially involved (anterior and proximal), but subsequent enlargement of these margins revealed no residual tumor. The final diagnosis was a vulvar LLT, and margins were negative after the wider excision. During a 24-month follow-up, no signs of recurrence were observed.

## Discussion

Primary mesenchymal tumors of the vulva are rare and mostly benign, with sarcomas accounting for approximately 1% of all malignant vulvar neoplasms. Among adipocytic tumors that may originate in this area, most are lipomas; however, vulvar liposarcomas represent less than 1% of all reported liposarcomas [7].

The LLT, as presented in this case, is a rare adipocytic neoplasm with a predilection for the vulva, although it has also been reported in other sites such as the scrotum, spermatic cord, and forearm [2]. Histologically, as observed, it consists of spindle cells in a myxoid stroma with prominent vasculature and variable adipocytic differentiation. This histology can lead to confusion with other lipomatous neoplasms, particularly myxoid liposarcoma, lipoblastoma, spindle cell lipoma, and atypical spindle cell/pleomorphic lipomatous tumor (ASCPLT) [3,8].

Recent findings in the molecular characterization of LLT

Recent molecular studies have shown that LLTs exhibit a simple genomic profile, characterized by a low mutational burden and few copy number alterations. A key finding is the absence of DDIT3 rearrangements, which helps differentiate LLT from myxoid liposarcomas, as seen in the present case. However, significant heterogeneity in the immunohistochemical expression of various markers has been noted, complicating precise diagnosis [2].

Regarding immunohistochemistry, CD34 and PLAG1 expression is variable, suggesting these markers are not definitive for diagnosis. Similarly, RB1 expression is inconsistent, with about half of the cases showing loss of expression, without a clear correlation to tumor location. DDIT3 expression is usually low and focal, with no evident relationship to genetic alterations in this gene [2].

Some cases have shown additional genetic alterations, including activating mutations in PIK3CA and MTOR, suggesting potential molecular pathways involved in tumor growth. Mutations in TSC1 and deletions in CDKN2A, CDKN2B, and MTAP have also been identified, although their clinical relevance remains unclear [2]. In this case, the absence of DDIT3 rearrangement and focal expression of CD34 and S100 are consistent with previously reported findings in LLT, supporting its classification within this tumor spectrum [2].

Although LLTs generally exhibit predominantly benign behavior, at least one case of pulmonary and pleural metastasis has been reported, where the metastatic tissue shared identical histological and molecular profiles with the primary tumor. This suggests that, in rare cases, LLT may have metastatic potential, highlighting the importance of long-term clinical follow-up, particularly in patients with persistent disease or incomplete resections. In the present case, extended follow-up was conducted, and the tumor exhibited typical behavior for LLT, with no evidence of metastasis [2,9].

Differentiation from other tumors

According to the 2020 WHO classification, LLT is defined by essential and desirable diagnostic criteria, which are summarized in Table 1. These criteria provide a framework for distinguishing LLT from other adipocytic tumors [6].

**Table 1:** WHO 2020 diagnostic criteria for LLT. LLT: lipoblastoma-like tumor; FISH: fluorescence in situ hybridization; IHC: immunohistochemistry. *Source*: Adapted from the WHO Classification of Tumors, 5th Edition [6].

Essential features	Desirable features
Lobulated growth pattern with fibrous septa of variable thickness	Absence of DDIT3 rearrangement
Mixture of mature adipocytes, univacuolated and bivacuolated lipoblasts, and bland spindle cells	Absence of PLAG1 rearrangement
Abundant myxoid matrix	RB1 gain or loss detected by FISH
Thin-walled branching vasculature	Lack of PLAG1 and HMGA2 expression in IHC

Histologically, LLT features spindle cells in a myxoid stroma with prominent vasculature and variable adipocytic differentiation, which can lead to diagnostic confusion with other adipocytic tumors such as myxoid liposarcoma, lipoblastoma, spindle cell lipoma, and atypical spindle cell/pleomorphic lipomatous tumor (ASCPLT), which are described below [3]. These differential diagnostic features are summarized in Table 2 [3,10,11].

**Table 2:** Differential diagnostic features of LLT and other adipocytic neoplasms. FISH: fluorescence in situ hybridization; ASCPLT: atypical spindle cell/pleomorphic lipomatous tumor; LLT: lipoblastoma-like tumor.

Tumor type	Cytopathology	Markers
LLT	Spindle cells in a myxoid stroma with prominent vasculature and variable adipocytic differentiation	Absence of DDIT3 and PLAG1 rearrangements; regional RB1 gain/loss detected by FISH
Myxoid liposarcoma	Myxoid stroma, plexiform vascular network, round to ovoid nuclei, uni- or multivacuolated lipoblasts with scalloped nuclei	CD34+, DDIT3 rearrangement
Lipoblastoma	Adipocytes, lipoblasts, spindle cells in clusters or isolated, vascular network, abundant myxoid material and naked oval nuclei	PLAG1 rearrangement
Spindle cell lipoma	Spindle cells with myxoid matrix, mature adipocytes, few lipoblasts	Strong diffuse CD34+, RB1 loss
ASCPLT	Spindle cells with infiltrative growth, frequent lipoblasts, marked nuclear atypia	Strong diffuse CD34+, RB1 loss, frequent additional deletions

Myxoid liposarcoma typically arises in the deep soft tissues of the extremities in young adults and shares some features with LLT, including a myxoid matrix and prominent plexiform capillary network. High-grade myxoid liposarcomas may exhibit nuclear atypia and high mitotic activity. However, they are composed of small round cells rather than spindle cells and contain multivacuolated lipoblasts, as opposed to the characteristic "signet ring" or "hourglass"-like lipoblasts seen in LLT. In difficult cases, molecular analysis for DDIT3 rearrangement is essential for differential diagnosis, as LLT lacks this alteration [3,10].

Lipoblastoma typically occurs in children and young adults, primarily affecting the extremities and trunk, and is characterized by prominent lobulated growth with mixed adipocytic differentiation, including both mature and immature adipocytes. Histologically, mature adipocytes are concentrated in the center of fibrous lobules. In contrast to LLT, lipoblastoma is associated with PLAG1 rearrangements. Although PLAG1 immunopositivity can be observed in some cases of LLT, it is not exclusive to lipoblastoma [3,10].

Spindle cell lipoma is most commonly found in the neck, shoulder, and back of middle-aged or older men, although it can also occur in the inguinogenital region. Histologically, it consists of uniform spindle cells arranged with bundled collagen, a myxoid matrix, and mature adipocytes, with few or no lipoblasts. Occasional lipoblasts are observed in approximately 50% of cases [12]. Unlike LLT, spindle cell lipoma exhibits minimal vascularization, which helps distinguish it from LLT. At the molecular level, spindle cell lipoma is associated with monoallelic or biallelic deletions of 13q13, affecting RB1, leading to loss of RB1 expression on immunohistochemistry. Although RB1 expression loss is also observed in LLT, genetic deletion of the *RB1* gene has not been detected in LLT, suggesting that alternative mechanisms, such as epigenetic regulation or protein degradation, may be responsible [11].

ASCPLT is a recently recognized entity that shares features with spindle cell lipoma and pleomorphic lipoma but differs due to its infiltrative growth, increased cellularity, marked nuclear atypia, higher frequency of lipoblasts, and evident mitotic activity. In contrast, LLTs do not exhibit significant infiltrative growth or mitotic activity. Immunohistochemically, ASCPLT demonstrates diffuse and strong CD34 expression, whereas LLT may test positive for CD34, but only focally and variably. Additionally, ASCPLT is positive for S100 and desmin. At the molecular level, ASCPLT shares RB1 loss with spindle cell lipoma but typically harbors additional deletions, such as RCBTB2 loss [11,13].

Clinically, LLTs present as a soft tissue mass with nodular components and, in some cases, associated pain or tenderness [11]. The definitive treatment is complete surgical excision, which generally results in an excellent prognosis despite the potential for recurrence. To date, only one case of metastasis has been reported. However, despite its dissemination, the lesion did not exhibit morphological features of malignancy and demonstrated a low proliferation rate [9].The main limitation in current knowledge is the scarcity of reported cases, which makes it difficult to establish the true biological behavior of LLT. In this context, long-term follow-up of patients with confirmed LLT is essential to better define recurrence patterns and clarify the potential for metastasis.

## Conclusions

In conclusion, LLTs should be considered in the differential diagnosis of myxoid adipocytic tumors, particularly in the vulvar region. Its accurate recognition, combined with a thorough clinical approach and long-term follow-up, will optimize patient management and help prevent unnecessary or inappropriate interventions.

## References

[1] Lae ME, Pereira PF, Keeney GL, Nascimento AG (2002). Lipoblastoma-like tumour of the vulva: report of three cases of a distinctive mesenchymal neoplasm of adipocytic differentiation. Histopathology.

[2] Anderson WJ, Mariño-Enríquez A, Trpkov K (2023). Expanding the clinicopathologic and molecular spectrum of lipoblastoma-like tumor in a series of 28 cases. Modern Pathol.

[3] Celeste G. Yergin, Michael Chang y Ryan M. Thomas (2022). When is a lipoma not a lipoma? Case report presenting a lipoblastoma-like tumor of the gluteal cleft in an older gentleman with literature review. ClinicalKey [Internet].

[4] Mirkovic J, Fletcher C (2015). Lipoblastoma-like tumor of the vulva: further characterization in 8 new cases. Am J Surg Pathol.

[5] Schoolmeester JK, Michal M, Steiner P, Michal M, Folpe AL, Sukov WR (2018). Lipoblastoma-like tumor of the vulva: a clinicopathologic, immunohistochemical, fluorescence in situ hybridization and genomic copy number profiling study of seven cases. Modern Pathol.

[6] Mirkovic J, Schoolmeester JK (2020). Female genital tumours. WHO Classification of Tumours, 5th Edition.

[7] Vásquez-Dongo C, Rivas A, Ferrer B (2022). Lipoblastoma-like tumor of the vulva: a case report and review of the literature (Article in Spanish). Revista Española de Patología.

[8] Val-Bernal JF, Hermana S, Sánchez R (2016). Intradermal lipoblastoma-like tumor of the lip in an adult woman. Actas Dermo-Sifiliográficas.

[9] Chia MR, Windsor M (2023). Metastatic lipoblastoma-like tumour. ANZ J Surg.

[10] Osama MA, Chatterjee P, Singh S, Pandey A, Mohta A (2025). Myxoid liposarcoma diagnosed on fine needle aspiration cytology: there is more to it than meets the eye. J Cancer Res Ther.

[11] Gross JM, Perret R, Coindre JM (2023). Lipoblastoma-like tumor and fibrosarcoma-like lipomatous neoplasm represent the same entity: a clinicopathologic and molecular genetic study of 23 cases occurring in both men and women at diverse locations. Mod Pathol.

[12] Bose KS, Sarma RH (1975). Delineation of the intimate details of the backbone conformation of pyridine nucleotide coenzymes in aqueous solution. Biochem Biophys Res Commun.

[13] Mariño-Enriquez A, Nascimento AF, Ligon AH, Liang C, Fletcher CDM (2017). Atypical spindle cell lipomatous tumor: clinicopathologic characterization of 232 cases demonstrating a morphologic spectrum. Am J Surg Pathol.

